# Cost-effective, personalized, 3D-printed liver model for preoperative planning before laparoscopic liver hemihepatectomy for colorectal cancer metastases

**DOI:** 10.1007/s11548-017-1527-3

**Published:** 2017-01-31

**Authors:** Jan Sylwester Witowski, Michał Pędziwiatr, Piotr Major, Andrzej Budzyński

**Affiliations:** 2nd Department of General Surgery, Jagiellonian University Medical College, Kopernika 21 St., 31-501 Kraków, Poland

**Keywords:** 3D printing, Preoperative planning, Laparoscopic surgery, Colorectal metastases, Hemihepatectomy

## Abstract

**Purpose:**

Three-dimensional (3D) printing for preoperative planning has been intensively developed in the recent years. However, the implementation of these solutions in hospitals is still difficult due to high costs, extremely expensive industrial-grade printers, and software that is difficult to obtain and learn along with a lack of a defined process. This paper presents a cost-effective technique of preparing 3D-printed liver models that preserves the shape and all of the structures, including the vessels and the tumor, which in the present case is colorectal liver metastasis.

**Methods:**

The patient’s computed tomography scans were used for the separation and visualization of virtual 3D anatomical structures. Those elements were transformed into stereolithographic files and subsequently printed on a desktop 3D printer. The multipart structure was assembled and filled with silicone. The patient underwent subsequent laparoscopic right hemihepatectomy. The entire process is described step-by-step, and only free-to-use and mostly open-source software was used.

**Results:**

As a result, a transparent, full-sized liver model with visible vessels and colorectal metastasis was created for under $150, which—taking into account 3D printer prices—is much cheaper than models presented in previous research papers.

**Conclusions:**

The increased accessibility of 3D models for physicians before complex laparoscopic surgical procedures such as hepatic resections could lead to beneficial breakthroughs in these sophisticated surgeries, as many reports show that these models reduce operative time and improve short term outcomes.

## Introduction

Although 3D printing technology has been introduced in surgical disciplines as an alternative tool for preoperative planning, it is still not routinely used. Most 3D models are currently constructed as pre-surgical tools in maxillofacial and orthopedic surgery [1], primarily due to low costs and the simple structure of the models. The use of 3D models is expected to be beneficial in general/gastrointestinal surgery. Pre-surgical guides are considered particularly helpful in procedures requiring highly accurate visualization of anatomy and can be more favorable in comparison to standard imaging techniques. Potential advantages include shorter operative time, recovery time [2, 3], reduced blood loss and better resection margins [3, 4]. 3D-printed models have also been shown to be more beneficial in preoperative planning than 3D-rendered images [5].

High cost is a major limitation in the application of three-dimensional (3D) printing to the practice of medicine [6, 7]. This is mainly due to the need for complex models consisting of more than one material type, to be proven useful in planning a demanding surgery. Very expensive (over $200,000 [8]) industrial-grade printers and an insufficient number of experts, familiar with both medical and technological concepts, have prevented healthcare providers from using 3D models in everyday surgical procedures.

We describe the development of a 3D-printed model of the liver of a patient with colorectal cancer metastasis using a cost-effective method, relatively inexpensive materials, commercial-grade printers and open-source and freeware software. Additionally, the methods described herein provide material control and precision comparable to the best current methods. To our best knowledge, this is the first full-sized liver model prepared with innovatory approach described in this article, which allows to create transparent liver models with use of low-cost fused deposition modeling (FDM) printing technique and silicone. So far, the only cost-effective liver models that have been generated have represented single hepatic vessels or portal vein, not showing them as a whole anatomy of the area [9]. Not parenchyma nor tumors have been shown in these early attempts of low-cost 3D liver modeling. Other applications of 3D printing in liver surgery have been based primarily on significantly less affordable PolyJet/MultiJet [6, 10, 15] or selective laser sintering (SLS) [13] techniques.

## Materials and methods

We present a case of a 52-year-old female patient (BMI 29.5 kg/m$$^{2})$$ who underwent laparoscopic colorectal resection (T3N1M0) for colonic adenocarcinoma with adjuvant chemotherapy. In follow-up computed tomography (CT) 2 years after primary resection a single metachronous metastasis has been found. The patient has been submitted to laparoscopic right hemihepatectomy. Prior to surgery a 3D model of the patient’s liver has been printed using the technique described below.

**Fig. 1:**
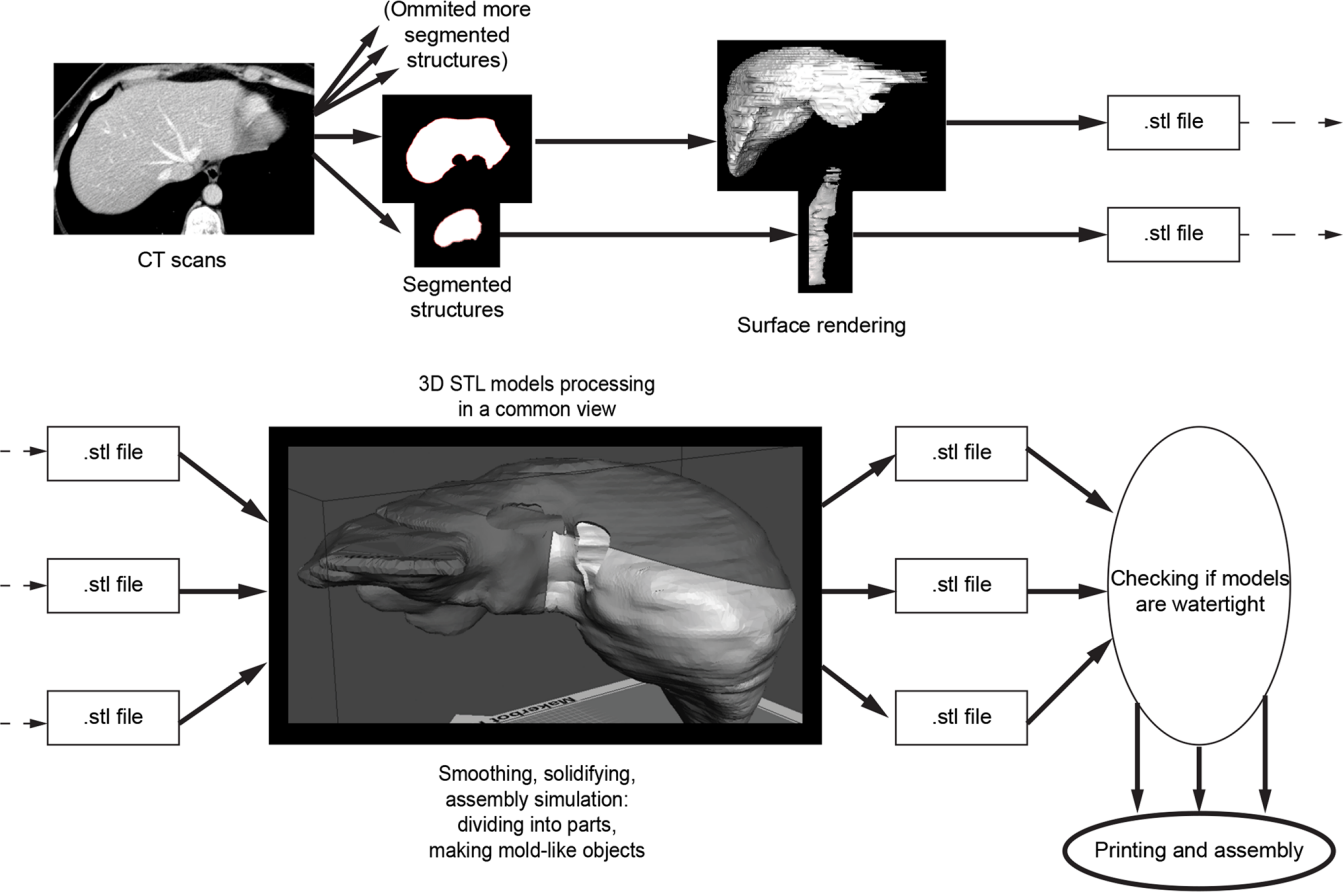
Workflow schema as described in the text. The key element of developing an approach was to process 3D models in a common view; this included dividing large virtual models into smaller, printable parts that were ready to assemble shortly after printing

The process of model development consists of four major phases: object segmentation, 3D model computer processing in common view, slicing and 3D printing, finishing and assembly with silicone curing (Fig. 1).

### Object segmentation

Raw contrast-enhanced, venous phase CT images (152 slices in one image series; scanned with Optima CT660, GE Healthcare; image size 512$\,\times \,$512 px; slice thickness 2.50 mm) were saved in a DICOM format. The images were then imported into open-source *Horos* software. The segmentation of anatomical structures was performed in a semi-automatic approach with pre-built functions available in the software. After using a region-growing, threshold-based algorithm, regions of interest (ROIs) were manually evaluated and corrected. Parameters for segmentation algorithms were chosen manually. Following segmentation, a 3D surface rendering was performed to verify segmented structures, and the desired models were exported as mesh-type, stereolithography (STL) files.

Mesh models were finalized using two free-to-use programs: Meshmixer (*Autodesk, Inc.*, San Rafael, CA, USA) and Blender (*Blender Foundation*, Amsterdam, Netherlands; open-source software). Processes that were performed using the software included the following: (1) the evaluation and removal of artifacts, i.e., overlapping vessel walls; (2) generating the model manifold (watertight); (3) adding thickness to the walls, which is a necessity in FDM printing; and (4) dividing the models into parts to fit the 3D printer dimensions and to prepare for silicone curing.

### 3D model processing in a common view

It is necessary to create multiple parts of one object when using this technique (for example, the liver parenchyma) to assemble the model, cast the silicone inside, and demount external parts afterward. Thus, during 3D processing in a common view (parallelly in the same coordinate system), multiple STL ready-to-print parts are created from one initial file, which are later printed and constructed into a model.

**Fig. 2:**
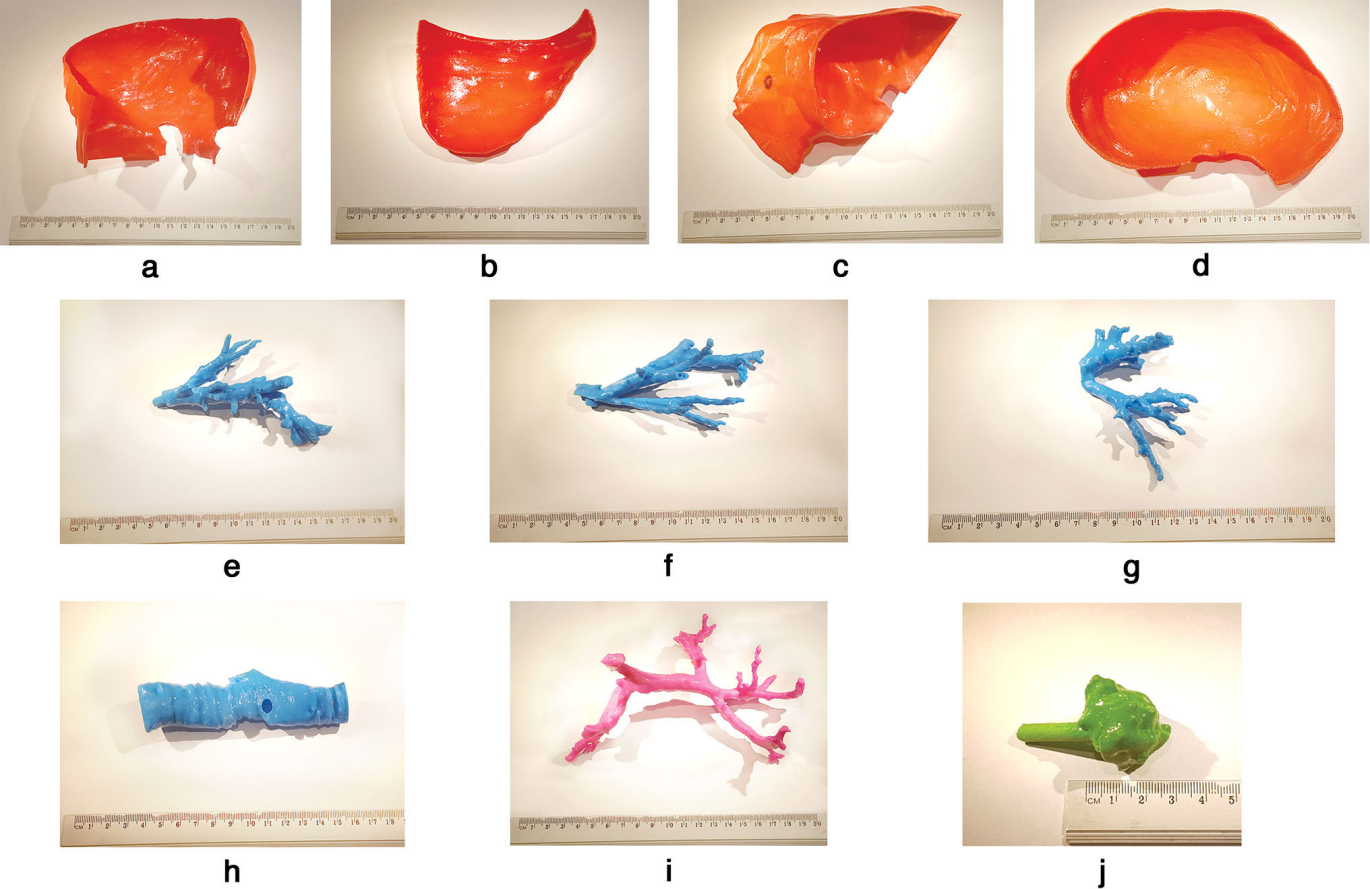
Physical parts of the 3D-printed liver model: 4 parts of the liver parenchyma (**a**–**d**) 3 parts of the hepatic veins (**e**–**g**), and the inferior vena cava (**h**). The portal vein (**i**) and the tumor with a connector as an assembly supporting element (**j**) are shown. Visible openings for the assembly of vessels on liver contour parts (**a**–**d**) can be seen; visible Boolean-based openings (**h**) and matching cylinder-shaped holders on vascular parts (**e**, **g**) are also shown. A 20 cm ruler is provided for scale below the models. Parts in the photos have undergone PLA postprocessing

Division was conducted in Blender and Meshmixer software programs; both allowed the execution of the same actions, and the choice was based on the user’s preference. Most importantly, vessels were split by a plane cut, with the plane located in the area in which they entered the liver parenchyma (Fig. 2e–g; hepatic vein separated from the inferior vena cava, Fig. 2h). To enable the connection of previously divided parts of the vascular tree, a cylinder was created at the side of the vessel fragment (Fig. 2e), and a matching opening (Fig. 2h) was formed at the other side using Boolean operations. Of note, both a plane cut and Boolean operations are basic functions in 3D modeling that are available in almost every designated software, and their use is straightforward. Those Boolean-based structures protect the correct location of every element after assembly.

Boolean difference operations were also executed on the external parts, and vessel parts that passed through the structure were subtracted from the external parts. This resulted in openings (see Fig. 2a–d) that allowed for assembly in later stages.

Liver contour-defining parts were then designated; in this case, they were divided into 4 segments. Planes of division passed through areas of vessels entering the parenchyma. This type of plane cut allowed the subsequent dismounting of the outer parts (Fig. 2a–d) to expose the final silicone model, which was crucial for the success of this technique.

The tumor, since it is usually not connected to any of the modeled vessels, must be attached to other available structures. A connector, shaped as an elliptic cylinder, was modeled and virtually unified with the tumor (Fig. 2j). Although the shape makes a visible difference, the supporting part can be painted after printing to avoid ambiguity of this solution.

It is necessary to divide the structure into smaller parts for simplicity or due to limitations of the 3D printer; slicing the element with a plane cut in a designated location is recommended. Both portions can later be reconnected using adhesive. In the present model, the middle and left hepatic vein were divided as described (Fig. 2f–g).

### Slicing and printing

Prepared models were sliced using open-source Cura (*Ultimaker*, Geldermalsen, Netherlands) software and printed using colored PLA with a desktop FDM Ultimaker 2+ 3D printer.

The total printing time can range from 60 to 100 h, depending on the size of the model, number of parts, printing accuracy, and type of printer. In this case, printing required approximately 72 h and was executed in 6 print jobs, due to interchanging material and build plate dimensions.

### Finishing PLA parts

The printed parts were subjected to a postprocessing stage to maximize the smoothness of the silicone surface. This is a necessary step that prevents cloudiness of the casted silicone parenchyma. First, all of the PLA parts of the liver parenchyma (inner sides) were sanded with 100–300 grit sandpaper. Sanded parts were washed with water and dried for several minutes. This was followed by coating with XTC-3D (*Smooth-On, Inc.*, Macungie, PA, USA), a self-leveling resin. Every part was covered in a thin layer and left to cure for approximately 3 h until the resin dried. Both steps (sanding and coating) were repeated once to ensure that the surface was sufficiently smooth for silicone casting.

If not processed, PLA liver parenchyma parts would appear significantly cloudy at the silicone surface, which would obscure all of the elements inside and render the model useless.

The inner elements (vessels and tumor) were coated with a thin layer of resin for additional cosmetic benefit only, since they do not affect model transparency.

### Assembly and silicone casting

**Fig. 3:**
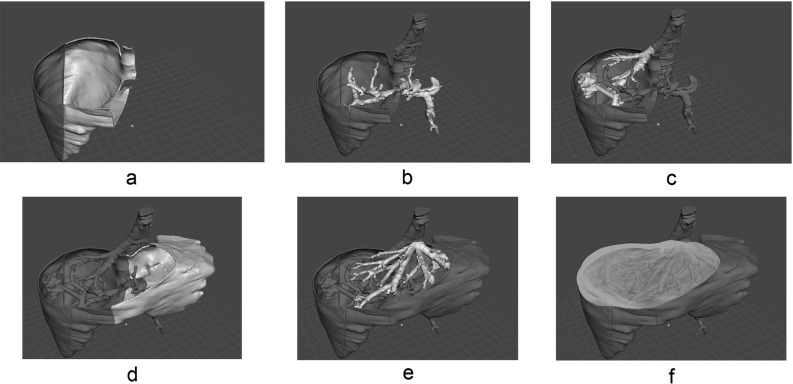
Assembly simulation. All of the parts in the 3D modeling software are shown in a common view. The goal is to determine the order of bonding with cyanoacrylate adhesive. In this study, the bonding order was as follows: **a** the two liver parenchyma parts; **b** the portal vein; **c** the inferior vena cava connected to the right hepatic vein and the tumor; **d** the third parenchyma part; **e** the left and middle hepatic vein; and **f** the fourth and final liver parenchyma part. This exact order was replicated during the assembly of the actual physical model

**Fig. 4:**
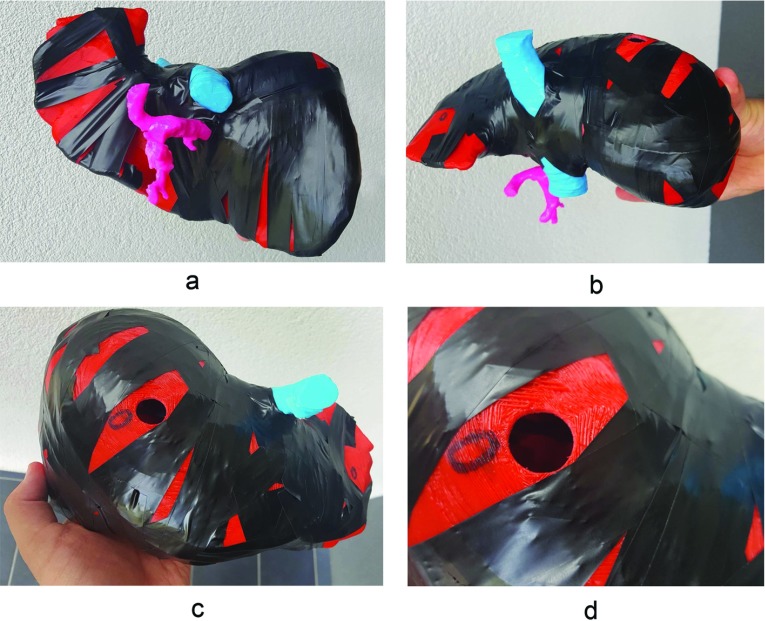
Assembly phase. All parts were connected with cyanoacrylate adhesive and secured with insulating tape and plasticine to prevent unevenness between connecting surfaces of the outer (*red*) parts and leaking of silicone (**a**, **b**). An opening was drilled at the top of liver model (**c**, **d**) to *insert* a funnel for the addition of silicone

The finished physical models (Fig. 2) were assembled as follows:

1. Multipart structures were glued together according to the assembly simulation (Fig. 3) using a common cyanoacrylate-based adhesive (known as Super glue$^{\textregistered }$). Orifices and matching cylinder-shaped filling parts were prepared using Boolean-based functions; this preserves the original location, rotation, and proportions of the entire structure after assembly.

2. After bonding all of the parts with cyanoacrylate adhesive, the model was additionally protected with insulating tape and plasticine in the connecting areas between parts. This step prevents silicone leakage during the silicone casting phase, and since it does not affect the model structure, it is safe to use (after this step the model appeared as shown in Fig. 4).

**Fig. 5:**
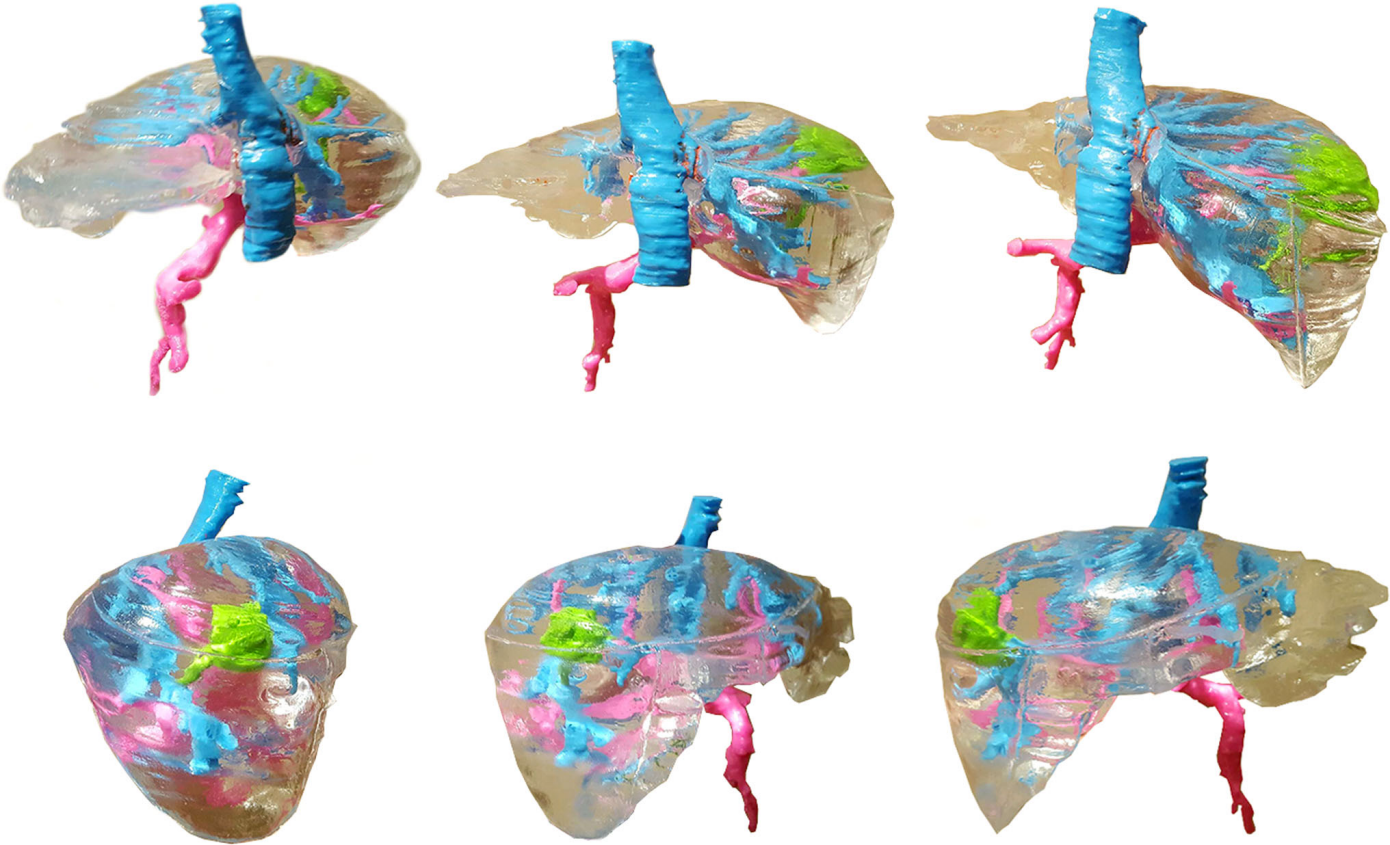
Photographs around fully complete liver model

3. Preparation of the silicone phase. Silicone rubber (Polastosil$${\textregistered }$$ M-2000, *Silikony Polskie Sp. zoo.*, Nowa Sarzyna, Poland) was hardened at room temperature using an OL-1 catalyst. This type of rubber is typically used to protect of electronic systems but can be used here due to its transparency and hardness. Silicone was manually mixed with the catalyst in a 100:7 proportion. Approximately 1250 g (1289 cm$$^{3})$$ of the mixture was needed to fill the liver model. The silicone density at 25 $$^{\circ }$$C was 0.97 g/cm$$^{3}$$, according to the manufacturer’s notes.
4. Silicone was poured inside the assembled model. There are several approaches to the execution of this step; a tube or a funnel can be placed in the model orifice and secured from leakage, or a “chimney” can be made at the top of the model. This step must be performed using the 3D modeling software before printing. The chimney approach is recommended since it is easy to prepare, even for beginners, and prevents problems with silicone leakage. In the described case, a hole was drilled at the top of the model that was large enough to fit the funnel (Fig. 4c, d).
5. The silicone was cured for 72 h at room temperature.
6. Removal of the liver parenchyma parts was performed carefully with a sharp tool, i.e., a sharp blade or a scalpel was used to cut through the adhesive in the linking areas while protecting the silicone from damage.

## Results

The final outcome of the process was a life-sized liver model with a transparent parenchyma, colorful plastic vessels and a tumor inside (Fig. 5).

The cost of production of a model similar to the one presented in this paper is estimated to be under $150 (Table 1). We estimate a total time of development, from segmentation to final object, to be around 160 h. Model was delivered to the surgical team scheduled to perform the procedure 5 days prior to the surgery. Surgeons had visually and tactilely inspected models and discussed the case, including the operative plan, with the aid of 3D model. The model was also available to the surgeon to be used as a intraoperative guidance tool.

The patient underwent fully laparoscopic right hemihepatectomy. The operative time was 270 min., intraoperative blood loss was 150 ml. Postoperative course was uneventful. Postoperative histopathologic evaluation confirmed R0 resection of the liver metastasis.

**Table 1:** Price estimate for the cost-effective liver model

Printing material (PLA, different colors, 3.00 cm thick)	$45
Silicone (rubber-type, with catalyst)	$35
Coating resin	$10
Tools (adhesive, insulating tape, sanding paper, plasticine,etc.)	Under $50

Labor costs and costs associated with 3D printer operation were neglected, since the authors did not require extra staff (experts, technicians, computer graphics, etc.) for execution

Models prepared with the described approach can be used to prepare surgeons for demanding procedures, such as laparoscopic resection of a colorectal cancer metastasis, in this case. Models would also be applicable for education of students and patients. The patient underwent subsequent hemihepatectomy.

## Discussion

This approach utilizes of one of the most affordable 3D-printing methods, FDM, to prepare complex models. Models using previous “standard” techniques had estimated prices of approximately $1000 for a liver model [10] and $500 for a kidney [11] model. Thanks to cost-effective approach, it seems possible to implement this solution in most hospitals worldwide. The technique can be executed by physicians after a relatively short training period.

3D-printed models are used in surgery to plan and prepare for extensive procedures, as a intraoperative guidance as well as an anatomy and pathology training tool [1]. Not only do models help surgeons, but they can also be used in many out-of-surgery applications, including education of students and young doctors in subjects such as anatomy, pathology, and surgery [12, 13] and in patient education [14]. All these make this low-cost method described in this paper feasible and justified among healthcare providers to implement 3D printing in clinical situations, which was previously available only to a limited number of hospitals.

Physical models also may be found more beneficial than 3D-rendered images. Virtual models do not represent structures in 1:1 scale, they also tend to be difficult to interpret in cases with complex anatomy, especially when evaluating the course of blood vessel branches. Although, there are only a few studies on this topic [5].

To date, PolyJet/MultiJet (photopolymer-based) or, more rarely, stereolithography (SLA) technologies have primarily been used to prepare similar models for pre-surgical preparation. However, PolyJet printers are industrial-grade, and their price varies from $6000 (low-end printers) to over $200,000 [8]. The high-end printers have been used to create pre-surgical anatomical models [11, 15]. Desktop 3D FDM printers are generally available starting from few hundred dollars and range up to $5000 for new-generation, relatively sophisticated FDM printers [16].

Accuracy of our model is affected by several factors. First, the resolution of the CT images most likely has the largest effect on the outcome. Some studies report that when a slice thickness of 2.5 mm is used, as in the present case, the liver volume may be underestimated [17]. However, semi-automated segmentation, as was presented in this paper, is reported to slightly overestimate the volume [18]. Computer processing and PLA finishing may have some effect on the volume, as some studies report 3D smoothing contributes to model shrinking [19], similar to coating PLA surfaces with thin layers of resin. Most of the described components mainly affect the parenchyma rather than the vessels or the tumor. Accurate calculations and dimension comparisons should be performed under conditions that allow for the measurement of the actual size of the liver. The ideal scenario for these measurements would be a transplantation surgery.

The accuracy of the model can be improved. For example, different algorithms can be utilized for segmentation, and CT scans with a lower thickness should be tested.

This technique requires creation of multiple parts of one object (for example, the liver parenchyma) to assemble the model, cast the silicone inside, and demount external parts afterward. Printing an entire model from one STL object would result in printouts filled with support material that would be impossible to remove. Additionally, it would not be possible to remove external parts to obtain the liver-shaped silicone parenchyma. This “multipart approach” also avoids limitations of the small printing field. Many 3D printers have printing field sizes smaller than 200$\,\times \,$200 mm, which is usually smaller than a full-sized liver. The full size models may be printed due to division. Many previous researchers describe the inevitability of scaling down models due to cost or printer limitations [10]. Moreover, smaller parts are usually easier to print (fewer artifacts and simpler removal of support material).

To date, few reports have described methods to correctly arrange multipart, complex structures, and some have reported the use of positioning joints and pins [20]. Our solution, using Boolean-based parts, is in our opinion accurate and does not disrupt the original structure.

Compared to significantly more expensive methods, our technique is slower (print time of 60–100 h compared to 36–40 h [6, 10]). However, the printing time can be reduced by half or more with the use of multiple printers, which still would be far more cost-effective. In addition, PLA printouts are more fragile than PolyJet-based printouts, especially during postprocessing. In addition, the assembly must be very accurate due to the risk of silicone leakage and the loss of model precision.

Liver malignancies surgery is not the only application of this method or of 3D-printed models. The relatively long printing time may limit the use of this technique in emergency surgeries; however, elective procedures, which represent the majority of hepatic resections [21], can greatly benefit from this 3D printing method.
